# Advancing Breast Cancer Diagnosis through Breast Mass Images, Machine Learning, and Regression Models

**DOI:** 10.3390/s24072312

**Published:** 2024-04-05

**Authors:** Amira J. Zaylaa, Sylva Kourtian

**Affiliations:** 1 Biomedical Engineering Program, Electrical and Computer Engineering Department, Faculty of Engineering, Beirut Arab University, Debbieh P.O. Box 11-5020, Lebanon; 2 Centre de Recherche du Centre Hospitalier, l’Université de Montréal, Montréal, QC H2X 0A9, Canada; sylva.kourtian.chum@ssss.gouv.qc.ca

**Keywords:** breast cancer, breast masses, medical imaging, fine-needle aspiration, diagnosis, artificial intelligence, machine learning, regression

## Abstract

Breast cancer results from a disruption of certain cells in breast tissue that undergo uncontrolled growth and cell division. These cells most often accumulate and form a lump called a tumor, which may be benign (non-cancerous) or malignant (cancerous). Malignant tumors can spread quickly throughout the body, forming tumors in other areas, which is called metastasis. Standard screening techniques are insufficient in the case of metastasis; therefore, new and advanced techniques based on artificial intelligence (AI), machine learning, and regression models have been introduced, the primary aim of which is to automatically diagnose breast cancer through the use of advanced techniques, classifiers, and real images. Real fine-needle aspiration (FNA) images were collected from Wisconsin, and four classifiers were used, including three machine learning models and one regression model: the support vector machine (SVM), naive Bayes (NB), k-nearest neighbors (k-NN), and decision tree (DT)-C4.5. According to the accuracy, sensitivity, and specificity results, the SVM algorithm had the best performance; it was the most powerful computational classifier with a 97.13% accuracy and 97.5% specificity. It also had around a 96% sensitivity for the diagnosis of breast cancer, unlike the models used for comparison, thereby providing an exact diagnosis on the one hand and a clear classification between benign and malignant tumors on the other hand. As a future research prospect, more algorithms and combinations of features can be considered for the precise, rapid, and effective classification and diagnosis of breast cancer images for imperative decisions.

## 1. Introduction

**Figure 1:**
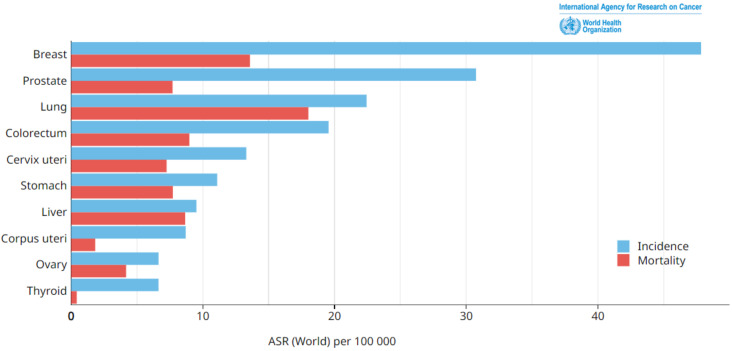
Estimated age-standardized cancer incidence and mortality rates issued by the World Health Organization (WHO) in 2020 (world, both sexes, all ages).

**Figure 2:**
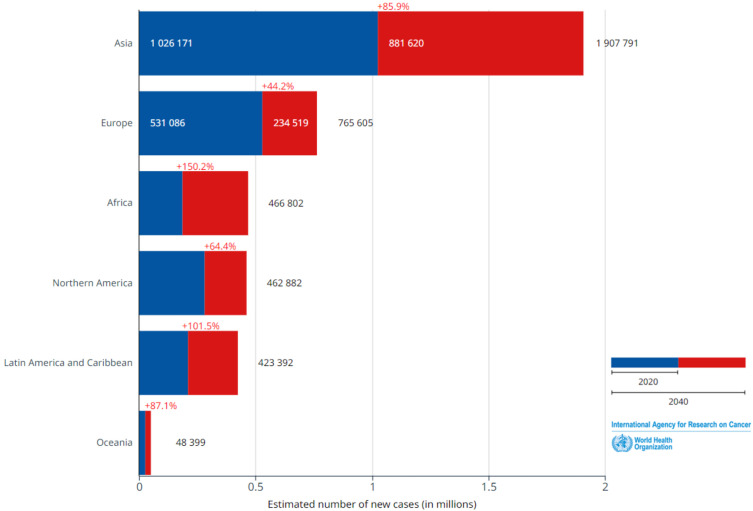
Estimated number of new breast cancer cases from 2020 to 2040 for both sexes and for ages 0–85+ years.

Breast cancer occurs when cells in breast tissues undergo uncontrolled growth and cell division. Breast cancer is classified into various types depending on the type of breast cell that becomes cancerous. According to statistics, breast cancer is considered the most predominant deadly disease globally, affecting both males and females, with a particular emphasis on women, and leading to significant mortality and suffering on a worldwide scale. An examination of the incidence and mortality rates issued in 2020 by the World Health Organization’s (WHO’s) International Agency for Research on Cancer revealed that breast cancer exhibited the highest occurrence among the various forms of cancer, as shown in Figure 1. The incidence of breast cancer in 2020 was estimated to be around 2,261,419 cases [1]. This organization’s studies indicate that this number is expected to rise significantly by 2040, reaching an estimated 4,074,871 new cases. This represents an alarming increase of 80%, as illustrated in Figure 2.

Considering the projected increase in breast cancer cases by 2040 [1], it is crucial to address the issue of the late detection of breast cancer and its associated effects. Minimizing the impacts of late detection and diagnosis on patients requires the prioritization of early detection. To accomplish this, it is crucial to use the complete potential of medical imaging techniques and their classification to aid in the early and automatic detection of cancer [1,2,3,4].

An article citing the International Agency for Research on Cancer (IARC), which reports to the WHO, has caused panic in Lebanon. According to this text, the prevalence of the disease in the country of the Cedar would be the highest among the countries of the Eastern Mediterranean; breast cancer remains the most common tumor in women in Lebanon with 2473 cases, according to the 2015 National Register, making up for nearly 37% of all female cancers. Of all reported cases, 36% of women with breast cancer were under the age of 50 years [1]. Another study conducted by researchers at a comprehensive medical center in Beirut showed that more than half of the Lebanese women with breast cancer were less than 50 years old. Based on these significant national statistics, it is judicious to study the early detection of breast cancer.

There are several diagnostic methods for breast cancer, including (1) breast self-examination (BSE), which consists of regular self-examination of the breast tissue and the detection of the presence of lumps or abnormalities; (2) the assessment of risk factors, such as age, family history, reproductive history, hormonal factors, and lifestyle factors, to determine an individual’s likelihood of developing breast cancer; (3) the evaluation of symptoms, such as breast pain, nipple discharge, changes in breast size or shape, and skin changes; and (4) the assessment of breast density, such as that obtained though medical imaging, (e.g., ultrasound imaging [5], mammography, and so on), as well as clinical assessments. Although ultrasound imaging is considered a safe imaging method [5], it cannot capture microcalcifications—tiny calcium deposits that can often be some of the earliest signs of breast cancer. Mammography is one of the imaging techniques used to detect breast abnormalities [6]. Observing breast mammograms is one of the standard screening tools for deciding whether breasts contain cancer or not. However, while mammograms can reveal suspicious findings that require further investigation, such as abnormalities that might indicate cancer, they do not allow for a definitive conclusion regarding its presence. Therefore, several investigations are required for the diagnosis of cancer. Moreover, mammography is based on X-rays, which use ionizing radiation [6]; furthermore, this type of imaging is sometimes insufficient, especially if the quality of the mammograms provided by the machines is low [3].

To improve the quality of the images, image processing has been applied to mammograms, in order to overcome the problems associated with manual diagnosis through replacing it with automatic diagnosis [3]. The purpose of processing medical images is to extract useful information from the acquired images for classification and diagnosis, thereby revealing details that are too vague to perceive with the naked eye while avoiding the production of artifacts and/or false information. To achieve the latter, processing involves algorithms that make it possible to act on a digitized image, and one of the fundamental processes is segmentation [7]. Ciecholewski applied morphological segmentation and watershed segmentation to mammograms with 200 regions of interest (ROIs) [8]. Duarte et al. worked on 1000 ROIs in mammograms from the Digital Database for Screening Mammography (DDSM) [9] and obtained a more precise diagnosis than that obtained in the research by Ciecholewski [8]. Although Duarte et al. and Arikidis et al. had previously worked on the segmentation of mammograms, Ciecholewski had higher overlap values (OVs) for microcalcifications [8,9,10]. Moreover, two types of microcalcifications were analyzed in the work of Ciecholewski—namely, those that are symptoms of malignant cases and those that represent benign cases—and fatty breast cases were generally considered [8]. Unfortunately, as the manual tracing of individual microcalcifications is very time-consuming for experts, taking almost 30 min for a single ROI, this forms an obstacle for conducting a larger number of experiments. Despite it being time-consuming, several researchers have used segmentation [7,8,9,10]. In addition, according to Ciecholewski, the mean sensitivity in all of the experiments amounted to 80% and reached a maximum value of 81% [8]. Therefore, the following question arose: is the segmentation of mammograms sufficient to automatically diagnose large data sets? Is it significant to employ artificial intelligence (AI)?

As machine learning (ML) algorithms, such as the support vector machine (SVM), naive Bayes (NB), k-nearest neighbors (k-NN), and decision tree (DT), have been used for the detection and classification of several events and diseases [4,11,12,13,14], similar algorithms could analogously be used for breast cancer classification [15]. Different research on the classification of breast cancer has used different models, such as SVM, multi-layer perceptron (MLP) model, other neural networks, and logistic regression [16,17,18]. Although, some models such as SVM have been used to detect breast cancer, where either ultrasound or mammography images were utilized, which do not provide information at the cellular level and thus required additional investigations for decisions.

Basically, the aim of this study was to find a harmony between image processing and AI techniques in order to provide an appropriate diagnosis during all phases associated with the detection of breast cancer, allowing for the construction of an automatic paradigm for breast cancer diagnosis. This was achieved through testing four classifiers—three ML algorithms and a regression model—for the diagnosis of breast cancer. In addition, this was achieved through calculating statistical metrics and the root absolute error (RAE) to determine the optimal classifier yielding an automatic diagnosis. Fine-needle aspiration (FNA) images and the cytology of classifications in the breast were used, as provided in the Breast Cancer Wisconsin (Diagnostic) Data set. FNA was used due to the fact that it is very challenging to detect breast abnormalities in mammograms with dense breast tissue. The proposed automatic and diagnostic paradigm could play a vital role in reducing diagnostic errors, providing physicians with an automatic and accurate diagnosis and promoting healthcare services, especially when numerous images are involved.

This article is divided into five main sections: After the introduction of the research context and recent studies related to breast cancer detection and diagnosis, Section 2 provides the experimental materials and methods used here, including the collection of experimental data, a priori information, features, classifiers, and evaluation methods. Section 3 showcases the results, which are divided into quantitative and qualitative results. Then, Section 4 discusses the results and provides a judicious conclusion. Finally, Section 5 opens the door for future points that can be considered in the automatic diagnosis of breast cancer.

## 2. Materials and Methods

Several medical imaging techniques are primarily utilized for the identification of breast cancer. These techniques include mammography, breast ultrasound (US), breast magnetic resonance imaging (MRI), computed tomography (CT), positron emission tomography (PET), and scintimammography. In addition, there are other techniques available—such as breast intervention procedures—that can aid in both the diagnosis and assessment of breast cancer [19,20]. Ongoing research aims to study and compare additional medical imaging techniques, highlighting their respective advantages and disadvantages. Comparative analyses that included the benefits and drawbacks of these techniques were reported by Iranmakani et al. and Iacob et al. [1,21]. Furthermore, various algorithms were developed to classify and determine the presence of breast cancer in medical images [3].

**Figure 3:**
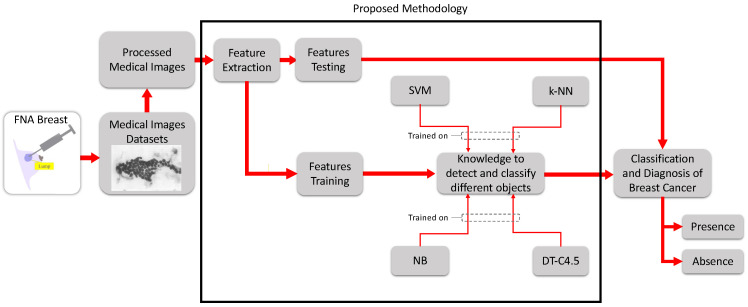
Block diagram of the proposed methodology for breast cancer diagnosis and evaluation.

This new paradigm, including the materials and proposed advanced methods used for the diagnosis and evaluation of breast cancer, is provided in Figure 3. Starting from the experimental data—fine-needle aspiration (FNA) images—and progressing to processing and feature extraction, a comparison of the performance of four classifiers—SVM, NB, k-NN, and DT-C4.5, which are among the most influential machine learning (ML) algorithms in medical research—was carried out. The goal was to assess the efficiency and effectiveness of these algorithms in terms of their accuracy, sensitivity, and specificity. The aim was also to calculate the error and measure of agreement of detection of breast cancer to evaluate the results, which were either reported as the “presence” of cancer (i.e., a malignant tumor) or the "absence" of cancer (i.e., a benign tumor).

To work on the paradigm shown in Figure 3, several materials were needed. First, the experimental data (i.e., FNA) were collected through a minimally invasive procedure performed on patients in a hospital and saved in the Wisconsin database [22]. Second, data processing and analysis were carried out through Matlab (R2022a, the MathWorks, USA) in the Biomedical Engineering Lab at the Faculty of Engineering at Beirut Arab University, Lebanon.

Pertaining to the proposed methodology, various procedures and steps were sequentially involved, as shown in Figure 3, starting from the collection of medical images of breasts, FNA data sets, and the decision-making process. The workflow is summarized as follows:

- First, the images were collected, imported, and prepared;
- Features were extracted from the images;
- Algorithms were trained;
- Algorithms were tested;
- The statistical evaluation metrics were assessed;
- The relative absolute error (RAE) and kappa statistic (KS) were calculated;
- The receiver operating characteristic (ROC) curves were plotted.

In the following, a detailed description of the database is provided.

### 2.1. Database Description

**Figure 4:**
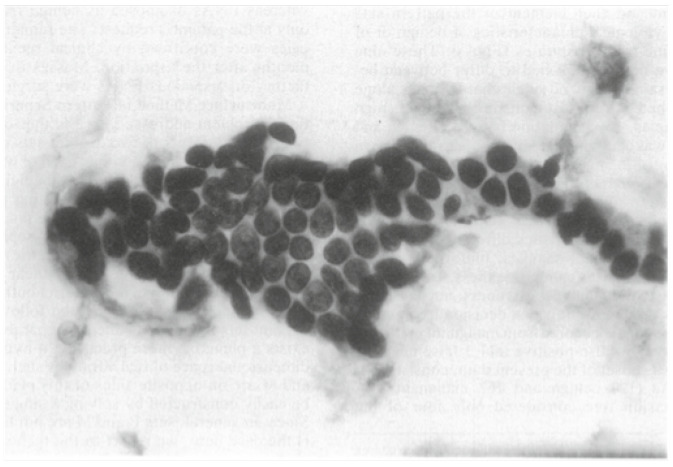
Identifying breast cancer in the fine-needle aspirate (FNA) of a breast mass.

A sample of the FNA of a breast mass (Figure 4) indicates the types of images that were employed in this study. FNA of the breast is a minimally invasive procedure used to diagnose breast abnormalities, such as lumps, cysts, and suspicious masses. With FNA, skilled healthcare professionals insert a small needle into the breast, taking samples of cells or fluid for further analysis. FNA is a diagnostic tool that helps to determine whether breast abnormalities are malignant (cancerous) or benign (non-cancerous); that is, there are two classes. Such early classification aids in the development of appropriate treatment plans. The FNA image shown in Figure 4 exhibits the characteristics of the cell nuclei. The database class distribution was composed of 357 benign and 212 malignant images.

After collecting the sample image shown in Figure 4, several features were extracted from it. A total of 569 digitized images of FNA of breast masses were processed.

### 2.2. Diagnosis and a Priori Knowledge

Diagnosis using FNA is usually based on a pathology report to evaluate a lump or mass in a patient’s breast as malignant or benign. Concerning a priori knowledge, nine cytological characteristics of breast FNAs were employed. The values of the cytological characteristics ranged from 1 to 10, with 1 being the closest to benign and 10 being the most malignant. Then, the data and a priori knowledge were categorized into the cancerous and non-cancerous categories. The a priori knowledge was employed, and 10 real-valued features were extracted from the FNA images.

### 2.3. Feature Extraction

Several features were thought of, including the following real-valued geometrical and non-linear features that were computed for each cell nucleus (as such, they are called nuclear features):

1. Radius, which signifies the mean of distances from the center to points on the perimeter;
2. Texture, which reflects the standard deviation of grayscale values;
3. Perimeter, which is the total length around the outside of a cell nucleus;
4. Area, which is the measure of the cell nucleus region’s size on a surface;
5. Smoothness of the nuclear contour, which reveals the local variation in radius lengths;
6. Compactness of the cell nuclei, which was calculated as (perimeter^2^/area − 1.0);
7. Concavity, which indicates the severity of concave portions of the contour;
8. Concave points, which illustrates the number of concave portions of the contour rather than the magnitude;
9. Symmetry, for which the longest chord through the center was found; then, the length difference between the lines that were perpendicular to the major axis to the cell boundary in both directions was measured;
10. The fractal dimension of a cell was approximated using “coastline approximation − 1”.

Moreover, the mean (M), standard error (SE), and worst (W) or largest (mean of the three largest values) of these features were computed for each image, resulting in 30 features. In addition to the 10 real-valued features, 2 other categorical variables were provided—the ID of each patient and the class label (malignant or benign).

Then, the features were input to train the classifiers, and some of them were used to test the classifiers after training was accomplished. Of the available data set (N = 569 (100%)), 70% of the data was used for training and 30% was used for the testing and validation of the classifiers. This showcased the distribution of the training, testing, and validation data sets for the diagnosis of breast cancer; A total of 357 images were benign and 212 images were malignant.

### 2.4. Classifiers

Four main classifiers were used: three ML algorithms and one regression algorithm.

#### 2.4.1. Support Vector Machine (SVM)

**Figure 5:**
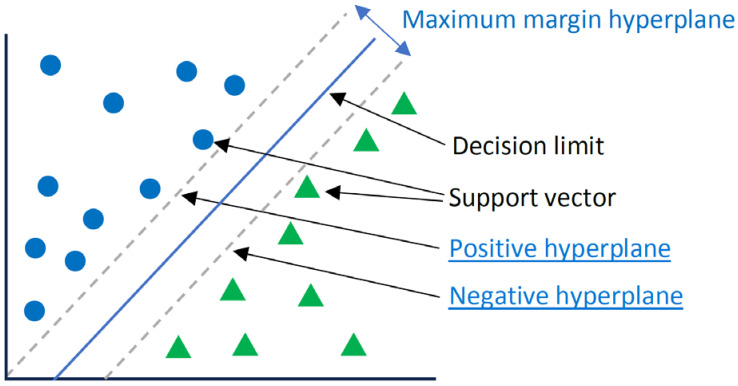
Parameters that delimit the hyperplane.

The SVM, which is also called a wide-margin separator, is a supervised ML algorithm [23,24]. SVMs are widely used in classification applications. SVMs are based on finding a hyperplane that best divides a data set into two classes. The hyperplane is a line separating and linearly classifying a set of data [23], and support vectors are the data points closest to the hyperplane. For instance, the data points shown in Figure 5 are classified in binary and are separated into two different classes. A decision boundary can be thought of as a dividing line where positive examples exist on one side, and negative examples exist on the other side. On this same line, data points can be classified as positive or negative. The parameters of the SVM used are as follows:

- The C parameter adjusts the margin and misclassification. A C parameter of 0.1 was chosen based on empirical inference, such that, a large C value meant less misclassification and a low margin. However, a high C value meant more misclassification but a large margin;
- The kernel parameters are functions used to calculate hyperplanes. As the data were separable with a linear line, a linear kernel was used. It was the simplest method for calculating hyperplanes.

#### 2.4.2. Naive Bayes (NB)

NB is a classification technique based on Bayes’ theorem with a hypothesis of independence among predictions [25]. The NB classifier assumes that the presence of a particular characteristic in a class is not related to the presence of another characteristic. For instance, a fruit can be considered an apple if it is red, round, and about 3 inches in diameter. Although these characteristics depend on each other or on the existence of other characteristics, all of these properties independently contribute to the probability that this fruit is an apple, and that is why it is said to be “naive”. NB models are easy to build and are particularly useful for very large data sets. This algorithm is mainly used in text classification and with problems that have multiple classes [26].

In addition to its simplicity, NB is known to surpass very sophisticated classification methods. Bayes’ theorem allows us to calculate the posterior probability P(c|x) from P(c), P(x), and P(x|c), as shown in Equation (1):

(1) $$P\left(c|x\right)=\frac{P(x|c)P(c)}{P(x)}$$

(2) $$P\left(c|x\right)=P\left(x_{1}|c\right)*P\left(x_{2}|c\right)*P\left(x_{3}|c\right)*...*P\left(x_{n}|c\right)*P\left(c\right).$$

where:

- P(c|x) is the posterior probability of a class (c, target) given the predictor (x, attributes);
- P(c) is the a priori class probability;
- P(x|c) is the likelihood, which is the probability of the predictor of a given class;
- P(x) is the prior probability of the predictor.

The steps that were involved in the classification of breast cancer were as follows:

1. Conversion of the data set into a frequency table;
2. Development of a likelihood table by finding the posterior values for each class. The class with the highest posterior probability was the result of the prediction.

NB uses a similar method to predict the probability of a different class based on various attributes. This algorithm is mainly used for problems with multiple classes [26]. Herein, the default NB was used with the parameters shown in Equation (1) for the classification of two classes.

#### 2.4.3. k-Nearest Neighbors (k-NN)

**Figure 6:**
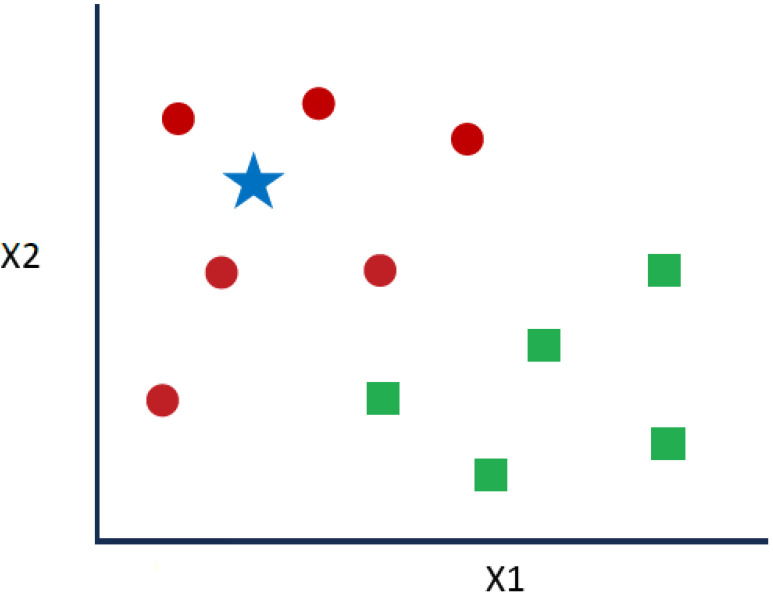
Example of the k-NN algorithm.

k-NN is used to classify target points (unknown class) as a function of their distances from points constituting a learning sample (i.e., the class of which is known a priori). k-NN is an intuitive supervised classification approach that is often used in the context of ML [27]. This is a generalization of the nearest neighbor (NN) method. NN is a special case of k-NN where k = 1. Given the series of red circles (RCs) and green squares (GSs) shown in Figure 6, if the intention is to know the blue star (BS) class, a BS can be an RC or a GS. The “k” in the k-NN algorithm denotes the considered number of closest neighbors.

**Figure 7:**
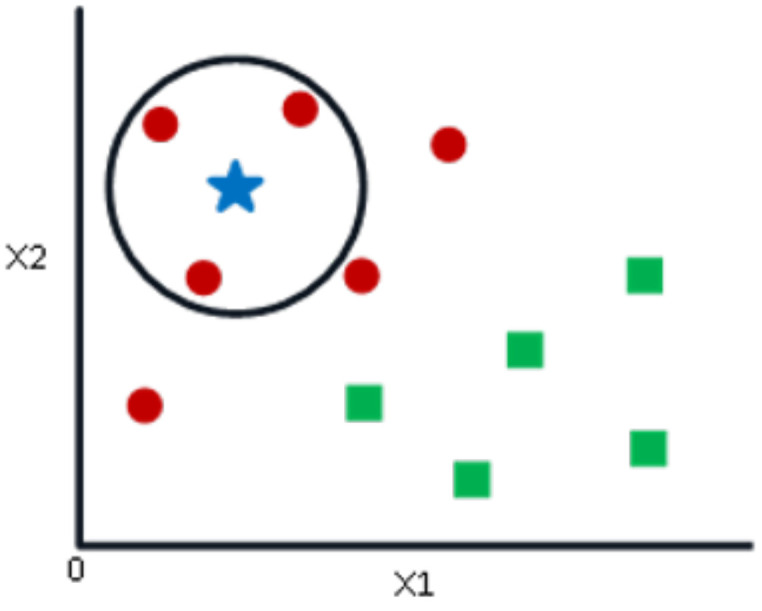
Example of the RC class.

If k = 3, a circle is drawn, with BS being the center, containing only three data points on the plan, as shown in Figure 7. The three closest points to the BS are all RCs, as shown in Figure 7. Thus, with a good level of trust, the BS belongs to the class RC. Here, the choice has become obvious as all three NN votes went to RCs. The choice of the parameter “k” is very crucial in this algorithm. Then, the factors are needed to decide the best value of k [21].

The parameters of k-NN used are as follows:

- The neighbors parameter k, which refers to the number of neighbors, was chosen to be 9 based on empirical inference;
- The weight parameter: unlike uniform weights, the distance of neighbors was considered as the weight showing the importance of classification;
- The algorithm parameter, which sets the algorithm that is used to compute the nearest neighbors;
- The p parameter, which refers to the distance between the query point and the neighbor, was chosen to be 1; that is, the Manhattan Distance, sum(|x−y|), was the best distance metric for the studied data set.

#### 2.4.4. Decision Tree (C4.5)

**Figure 8:**
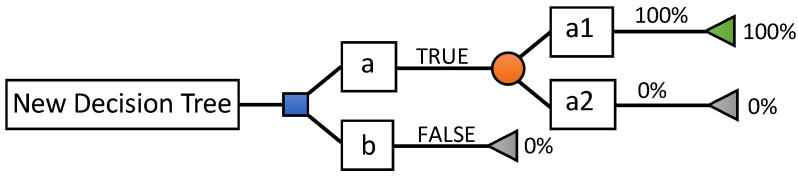
Decision tree elements [18].

DT is a regression technique in which the characteristics are independent of each other on a continuous basis. In graph theory, a tree is an undirected, acyclic, and connected graph [1]. DTs are a category of trees used in the exploration of data and business intelligence. They employ a hierarchical representation of a data structure in the form of sequences of decisions (tests) for the prediction of a result or class. Each individual (or observation), which must be assigned to a class, is described by a set of variables that are tested in the nodes of the tree. Testing takes place in internal nodes, and decisions are made in a node’s leaves. In decision analysis, a decision tree and influence diagram are used as a visual and analytical decision support tool, where the values’ expected (or expected utility) competing alternatives are calculated. As shown in Figure 8, a decision tree is made up of three types of nodes:

- Decision nodes, represented by squares;
- Lucky knots, represented by circles;
- End nodes, represented by triangles.

C4.5 was used to build a DT from a set of training data using the concept of information entropy. C4.5 builds a DT from a set of training data, and the training data are a set $W=w_{1},w_{2},\ldots$ of already classified samples. Each sample $w_{i}$ consists of a p-dimensional vector $\left(u_{1,i},u_{2,i},\ldots ,u_{p,i}\right)$, where $u_{j}$ represents attribute values or features of the sample, as well as the class in which $w_{i}$ falls.

After testing the classifiers, the results required specific methods for evaluation.

### 2.5. Evaluation Criteria

After applying the classifiers and in order to evaluate the results, the measurement that was the closest to the true value was expressed with the accuracy (i.e., the correctly predicted class over the total test class). This can be defined as the percentage of correctly classified instances as follows:

(3) $$Accuracy=\frac{\left(TP+TN\right)}{\left(TP+TN+FP+FN\right)}$$

where TP represents the number of true positives, FN represents the number of false negatives, FP represents the number of false positives, and TN represents the number of true negatives [28]. In addition, the following standard performance measures were also used to evaluate the results:

(4) $$Sensitivity=\frac{TP}{\left(TP+FN\right)}$$

and

(5) $$Specificity=\frac{TN}{\left(TN+FP\right)}.$$

Furthermore, to better understand the efficiency of detection and diagnosis, the ROC curve was simulated, providing a graphical representation of the relationship between the sensitivity and specificity of a test for all possible cutoff values [26]. The ordinate of the ROC represents the sensitivity (TP rate), and the abscissa corresponds to a 1-specificity quantity (FP rate).

In addition to the accuracy, sensitivity, and specificity, the simulation error and kappa score (KS) were also computed to reflect the effectiveness of the classifier as follows:

- The relative absolute error (RAE) is expressed as a ratio comparing the mean error (residual) to errors produced by a naive model. The RAE is defined as:
  (6) $$RAE=\frac{\sqrt{\sum_{i=1}^{n}{\left(x_{i}-y_{i}\right)}^{2}}}{\sqrt{\sum_{i=1}^{n}{\left(y_{i}\right)}^{2})}}$$
  where *y* is the actual result and *x* is the predicted result. A reasonable model (one that produces results that are better than those of a trivial model) results in a ratio of less than one [29];
- KS is a randomly corrected measure of agreement between classifications and real classes. The percentage of kappa values ≤ 0% indicate no agreement, 1–20% indicates no to slight agreement, 21–40% indicates fair agreement, 41–60% indicates moderate agreement, 61–80% indicates substantial agreement, and 81–100% indicates almost perfect agreement [30].

## 3. Experimental Results

**Figure 9:**
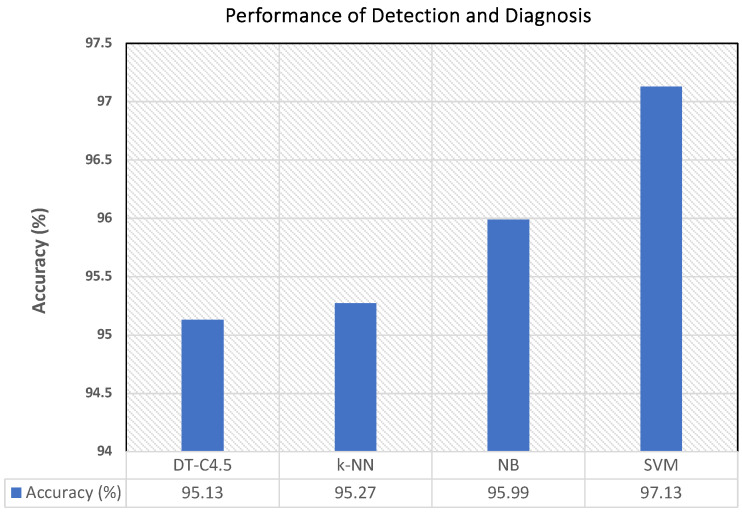
The statistical performance, accuracy of detection, and diagnosis of breast cancer using the four different classifiers: SVM, NB, k-NN, and DT-C4.5.

In order to measure the effectiveness of the automatic diagnosis of breast cancer achieved with the explored classifiers (SVM, NB, k-NN, DT-C4.5), qualitative and quantitative results are provided. The accuracy of diagnosis is reported and shown in Figure 9, where the graph depicts the behavior of the percentage of accuracy of the diagnoses obtained with the four different classifiers. The highest accuracy was obtained with SVM, followed by NB, k-NN and, finally, DT-C4.5.

Then, the effectiveness of diagnosis was tested based on the TP and FP results in comparison with the accuracy metric, as shown in Table 1 for SVM, NB, k-NN, and DT-C4.5. It is noteworthy that, depending upon the cancer type, the cells have a variation in size and shape. Thereby, the uniformity in the cell size or shape tends toward benign classification; otherwise, it is considered malignant. These two categories are reported in the table. In addition, other parameters, such as bare nuclei, bland chromatin, and normal nucleoli, are signs of benign masses. Table 1 shows that the highest value of TP (highlighted in bold) was 97%, and the percentage of diagnoses between malignant and benign cases was above or equal to 91% among all of the classifiers. In addition, for all of the classifiers, the FPs were inferior to 1%. The minimum values of FPs for the malignant and benign cases were obtained with the SVM and then NB approaches.

In addition to the accuracy, TP, and FP values, the sensitivity and specificity results of automatic diagnosis are reported in Table 2. Although the sensitivity of detection with NB (96%) dominated those of both the k-NN (94%) and DT-C4.5 (94.5%) algorithms, it was still approximately comparable with that obtained with the SVM.

For the specificity of diagnosis, the result obtained with the SVM was 97.50% and was larger than that obtained with NB (97%), k-NN (95%), and C4-5 (95.5%). Therefore, both the accuracy and specificity values of SVM were better than those of the other ML algorithms used and had the lowest error rate.

**Figure 10:**
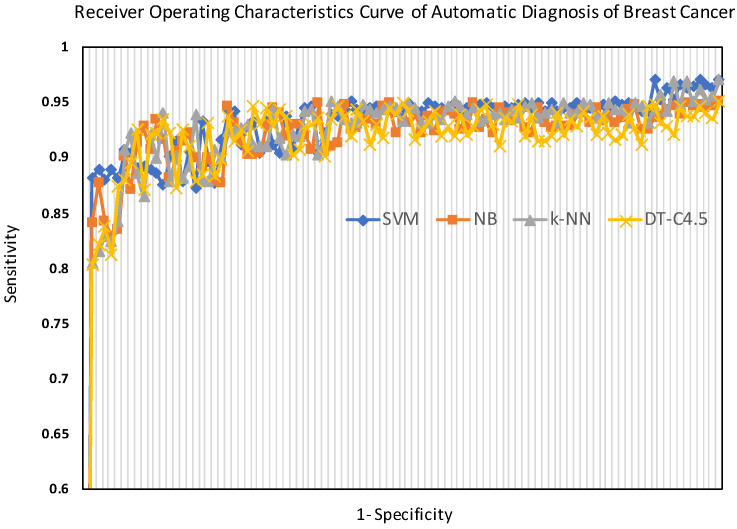
The receiver operating characteristic (ROC) curves of the support vector machine (SVM, highlighted in blue), naive Bayes (NB, highlighted in orange), k-nearest neighbor (k-NN, highlighted in gray), and decision tree-C4.5 (DT-C4.5, highlighted in yellow) for breast cancer diagnosis.

Moreover, the ROC curves of the tested classifiers (SVM, NB, k-NN, and DT-C4.5) are shown in Figure 10. The ROC curves, shown in Figure 10, vividly illustrate the performance of the classifiers, showing that the SVM approach provided the best diagnosis.

**Figure 11:**
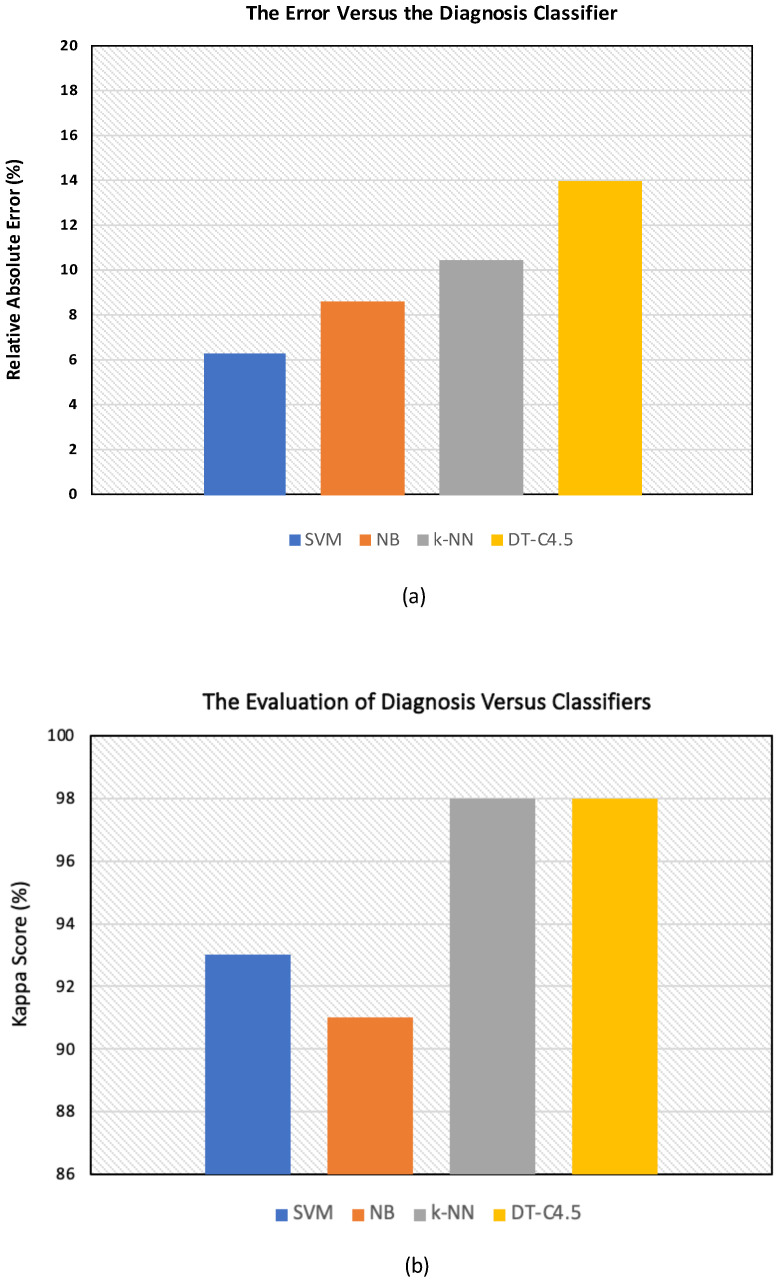
The bar graphs of the machine learning algorithms and regression algorithm in the diagnosis of breast cancer. (**a**) The errors of diagnoses yielded by the four classifiers. (**b**) The kappa score (KS) of diagnoses yielded by the four classifiers.

Furthermore, the training and simulation errors of diagnosis yielded by the four classifiers are reported in Figure 11a,b. It is noteworthy that the RAEs are reported in percentages (%) in Table 3 and are shown in Figure 11a. Moreover, the KS values are also reported in percentages (%) in Table 3 and are shown in Figure 11b.

Regarding the measurement of the error, according to Table 3, the chance of having a significant classification (93%) with the lowest relative absolute error (RAE) was obtained with the SVM, with the %RAE being 6.33%.

## 4. Discussion and Conclusions

This study explored the application of three distinct ML algorithms—namely, SVM, NB, and k-NN—as well as the DT-C4.5 regression model for the classification and diagnosis of breast cancer. The imaging data set utilized in this study was collected, processed, and subsequently employed for training. The final determination of the presence and/or absence of cancer was performed with specific classifiers selected from the aforementioned algorithms.

It is noteworthy that the accuracy obtained with the SVM classification was better than that obtained with C4.5, NB, and k-NN. The accuracy values varied between 95.13% and 97.13% in our work.

Based on these outcomes, in order to discuss the results and compare the performance in diagnosis, further results are reported in Table 4, taking the SVM as a reference and checking all of the results based on the SVM.

The results show that each employed algorithm detected breast cancer with a relatively high accuracy and well-defined efficiency. The SVM was the most efficient classifier in the diagnosis of breast cancer based on the outcomes, showcasing an ultimate accuracy of 96.84%, the greatest specificity of 97.36%, and the greatest sensitivity of 95.85%; this high performance of the SVM was in accordance with that reported in medical-related studies [31]. However, the difference in the performance of the algorithms should be noted by taking the SVM as a reference, as shown in Table 4. The minimum difference in the absolute performance is highlighted in bold for each statistical measure explored. The minimum difference values showed an overall close performance between NB and the SVM.

However, the difference in the absolute error distribution was at its maximum between the SVM and DT-C4.5; therefore, the error distribution was minimal for the SVM and maximal for DT-C4.5 (SVM<NB<k−NN<DT−C4.5). In addition, the difference in the accuracy distribution was as follows: Δ(SVM,DT)>Δ(SVM,k−NN)>Δ(SVM,NB). Therefore, the maximum accuracy distribution was as follows: SVM>NB>k−NN>DT−C4.5. Furthermore, the difference in the sensitivity distribution was as follows: Δ(SVM,k−NN)>Δ(SVM,DT)>Δ(SVM,NB). Hence, the maximum sensitivity distribution was as follows: SVM≃NB>DT−C4.5>k−NN. In addition, the difference in the specificity distribution was as follows: Δ(SVM,k−NN)>Δ(SVM,DT)>Δ(SVM,NB). Therefore, the maximum specificity distribution was as follows: SVM>NB>DT−C4.5>k−NN. This difference in percentages is due to the differences in the theory and principle of operation of each algorithm.

In addition, ML classification (SVM, NB, k-NN) surpassed the classification with the regression algorithm (DT-C4.5). This could be due to the fact that the DT algorithm is known for its ability to handle complex non-linear relationships in data [1]; however, the currently studied data did not totally possess non-linear relationships. Moreover, the highest performance of the SVM was due to the fact that SVMs are effective at finding the optimal margin of separation in high-dimensional space.

Despite the advantageous outcomes of the SVM, it can be more computationally intensive for larger data sets; hence, DT-C4.5 can be faster, which is in accordance with the outcomes of Iacob et al. [1].

By comparing the accuracy of diagnosis of breast cancer in our study with that obtained by other researchers [17,18,22], the accuracy obtained with the SVM was 2.7% higher than that obtained and reported by Obaid et al. (94.4%), as shown in Table 5. This could be due to the difference in the choice of kernel function.

Furthermore, the accuracy obtained with our paradigm using NB was higher than that obtained by Kharya et al. (93%) [32]. This could be due to the fact that Kharya et al. applied their model to the Wisconsin prognostic database rather than the diagnostic one, and they used the model to predict cancer.

In addition, the SVM’s accuracy of diagnosis was 4.83% higher than that obtained with the neural network (92.3%) and 1.33% higher than that obtained through logistic regression (95.80%) [17,18]. This could also be due to the fact that the data set was labeled with two classes: malignant and benign.

Furthermore, the accuracy obtained by Street et al. was 86% after using snakes customized to the exact shape of the nuclei [33]. Therefore, the results obtained in this work were better than those presented in similar research [33].

The results obtained with a multi-layer perceptron (MLP) showed that the efficiency of classification reached a maximum of 94% [16], but the outcomes of this study surpassed the outcome for MLP, as the accuracy of diagnosis ranged approximately between 95% and 97% when using the aforementioned database in the current work.

On the other hand, ML aided in the automatic classification of breast cancer and produced promising results when applied to a data set that was labeled as malignant and benign. This was in accordance with the general concept triggering the use of ML [4,31] as, usually, when doctors want to specify the type of tumor—whether it be malignant or benign—they use their own experience acquired throughout their career and study [3]; that is, they rely on their personal logic, mental effort, and personal intelligence to determine the diagnostic result, which is provided automatically to help them in the decision.

The major challenge of applying advanced algorithms to medical data is providing accurate and computationally efficient classifiers for medical purposes. In the current study, four algorithms—SVM, NB, k-NN, and DT-C4.5—were applied to breast cancer data sets. The performance of these algorithms was measured in terms of statistical measures, accuracy, sensitivity, and specificity to determine the best classification performance. The SVM classification achieved an accuracy of 97.13% and therefore outperformed all other algorithms. Compared with the fair amount of breast cancer research in the literature, using the Wisconsin data set to compare the classification accuracy of exploration algorithms, our experimental results provided the highest value of accuracy in classification.

Hence, the SVM proved its effectiveness in the prediction and diagnosis of breast cancer for good management and achieved the best performance with a low error rate.

Despite the method of operation, all of the employed algorithms were able to correctly diagnose breast cancer based on the images with a minimum error rate (RAE), which is related to quality control, referring to the technical act that makes it possible to determine the conformity and quality of an image when two quality parameters—true positives (TP) and false positives (FP)—are reported. These two parameters are able to confirm the presence of a pathology or its absence based on the following three assessment parameters: accuracy (percentages of cases that are diagnosed correctly), specificity or 1-FP (probability of obtaining a negative diagnosis in a patient who does not have breast cancer), and sensitivity or PT (probability of obtaining a positive diagnosis in a patient with breast cancer). Finally, a true diagnosis is provided. Previous studies explored the evolution of imaging techniques and their impacts [4,19]. These studies concluded that, as imaging techniques continue to advance, the collaboration among radiologists, surgeons, and ML algorithms will become more intertwined.

The originality of our findings was demonstrated according to the performance measures of diagnoses based on FNA images being the highest, as these images showcase the cells (i.e., at the cellular level), unlike ultrasound imaging, which shows the density at the tissue level; and mammography, which works on the attenuation of the ionizing radiation of X-rays.

Despite the advancement and effectiveness of automatic breast cancer diagnosis through imaging and classifiers, there remains a limitation regarding the number of FNA images used for feature extraction to be input into the classifiers; that is, as the number of images increases, the outputs of the classifiers and algorithms are augmented. In addition, in the current work, ML algorithms and a regression model were used in order to choose the optimal algorithm for the classification and detection of cancer, but the performance of deep learning algorithms can be further investigated in future research.

## 5. Future Work

As breast cancer continuously affects more women each year, early automatic detection through screening and prompt evaluation of any abnormalities are key factors in improving the outcomes for individuals with breast cancer. Although clinical methods provide significant initial information, they require further evaluation with imaging studies if abnormalities are detected. Thus, it is judicious to study advanced techniques for better diagnosis, such as studying the effectiveness of machine learning (ML) for the diagnosis of cancer of the breast through experimental and real FNA images at the cellular level. The new paradigm used, which included three ML models and one regression model that were applied to such images, validated our hypothesis that breast cancer diagnosis could be automatically and accurately achieved through integrating FNA images at the cellular level and ML and regression models. Moreover, regardless of the experience and evaluation of the physician, the statistical evaluation measures are promising and the error rate is low, making automatic diagnosis worthwhile. Thereby, the advancement of imaging techniques and their impact on patient diagnosis are driven by a shared objective of achieving the best possible outcomes for patients with breast cancer. Through optimizing imaging modalities to provide precise and comprehensive information that can effectively guide surgical interventions, the outcomes of the current work bring us closer to realizing this common goal.

Despite the advancements in medical breast cancer imaging, accurately classifying breast cancer remains challenging, particularly in early-stage cases or when dealing with dense breast tissues. Therefore, exploring FNA and breast mass images through the utilization of algorithms for precise, rapid, and effective classifications and diagnoses of medical images is imperative. However, it could also be useful to test our paradigm when considering images at different levels (i.e., those other than at the cellular level). Not only mammography and ultrasound images, but also additional algorithms and different combinations of features can be considered in future research. Furthermore, for malignant tumors, the degree of aggressiveness could be explored in future research.

## Figures and Tables

**Table 1:** The diagnosis results for the four classifiers—SVM, NB, k-NN, and DT-C4.5—based on the TP and FP measurements.

Algorithm	True Positive	False Positive	Class
**SVM**	**0.97**	0.03	Benign
	0.94	0.02	Malignant
**NB**	0.95	0.02	Benign
	**0.97**	0.04	Malignant
**k-NN**	**0.97**	0.08	Benign
	0.91	0.02	Malignant
**DT-C4.5**	0.95	0.05	Benign
	0.94	0.04	Malignant

**Table 2:** The sensitivity, specificity, and accuracy of automatic diagnosis using a support vector machine (SVM), naive Bayes (NB), k-nearest neighbor (k-NN), and decision tree (DT-C4-5).

Algorithm	Sensitivity (%)	Specificity = 1 − FP (%)	Accuracy (%)
Support Vector Machine (SVM)	95.85	97.50	97.13
Naive Bayes (NB)	96.00	97.00	95.99
k-Nearest Neighbor (k-NN)	94.00	95.00	95.27
Decision Tree (DT-C4-5)	94.50	95.50	95.13

**Table 3:** The evaluation metrics for the four employed classifiers: support vector machine (SVM), naive Bayes (NB), k-nearest neighbor (k-NN), and decision tree-C4.5 (DT-C4.5).

Classifiers	SVM	NB	k-NN	DT-C4.5
Kappa Score (KS) (%)	93.00	91.00	98.00	98.00
Relative Absolute Error (RAE) (%)	6.33	8.59	10.46	14.00

**Table 4:** The absolute difference Δ of the evaluation metrics for the NB, k-NN, and DT-C4.5 classifiers when taking the SVM as a reference.

Classifiers	i = NB	i = k-NN	i = DT-C4.5
ΔAccuracy (SVM-i) (%)	**1.13**	1.86	2
ΔSensitivity (SVM-i) (%)	**0**	2	1.50
ΔSpecificity (SVM-i) (%)	**0.50**	2.50	2
ΔRelative Absolute Error (RAE) (%)	**2.26**	4.13	7.67

**Table 5:** Comparative results of the statistical metrics for breast cancer diagnosis.

Method	Types of Images	Performance (%)
**New Paradigm (SVM)**	FNA, Breast Mass	Accuracy (**97.13**%)
**New Paradigm (NB)**	FNA, Breast Mass	Accuracy (**95.99**%)
**New Paradigm (k-NN)**	FNA, Breast Mass	Accuracy (95.27%)
**New Paradigm (DT-C4.5)**	FNA, Breast Mass	Accuracy (95.13%)
SVM by Obeid et al. [18]	FNA, Breast Mass	Accuracy (94.4%)
NB by Kharya et al. [32]	FNA, Breast Mass	Accuracy (93%)
Logistic Regression by Obeid et al. [18]	FNA, Breast Mass	Accuracy (95.8%)
MLP by Desai et al. [16]	FNA, Breast Mass	Efficiency (94%)
Neural Network by Obeid et al. [18]	FNA, Breast Mass	Accuracy (92.3%)
Snakes Customized Method by Street et al. [33]	FNA, Breast Mass	Accuracy (86%)

## Data Availability

Data are contained within the article.

## References

[1] Iacob R., Manolescu D.L., Stoicescu E.R., Fabian A., Malita D., Oancea C. (2022). Breast Cancer—How Can Imaging Help?. Healthcare.

[2] Zaylaa A.J., Makki M., Kassem R. Thalassemia Diagnosis Through Medical Imaging: A New Artificial Intelligence-Based Framework. Proceedings of the 2022 International Conference on Smart Systems and Power Management (IC2SPM).

[3] Mendes J., Domingues J., Aidos H., Garcia N., Matela N. (2022). AI in Breast Cancer Imaging: A Survey of Different Applications. J. Imaging.

[4] Zaylaa A.J., Wehbe G.I., Ouahabi A.M. Bringing AI to Automatic Diagnosis of Diabetic Retinopathy from Optical Coherence Tomography Angiography. Proceedings of the 2021 Sixth International Conference on Advances in Biomedical Engineering (ICABME).

[5] Anghelache Nastase I.-N., Moldovanu S., Moraru L. (2022). Image moment-based features for mass detection in breast US images via machine learning and neural network classification models. Inventions.

[6] Shah D., Ullah Khan M.A., Abrar M. (2024). Reliable Breast Cancer Diagnosis with Deep Learning: DCGAN-Driven Mammogram Synthesis and Validity Assessment. Appl. Comput. Intell. Soft Comput..

[7] Amin M.M., Tabatabaeian M., Chavoshani A., Amjadi E., Hashemi M., Ebrahimpour K., Klishadi R., Khazaei S., Mansourian M. (2019). Paraben content in adjacent normal-malignant breast tissues from women with breast cancer. Biomed. Environ. Sci..

[8] Ciecholewski M. (2017). Microcalcification segmentation from mammograms: A morphological approach. J. Digit. Imaging.

[9] Duarte M.A., Alvarenga A.V., Azevedo C.M., Calas M.J.G., Infantosi A.F., Pereira W. (2015). Evaluating geodesic active contours in microcalcifications segmentation on mammograms. Comput. Methods Programs Biomed..

[10] Arikidis N.S., Karahaliou A., Skiadopoulos S., Korfiatis P., Likaki E., Panayiotakis G., Costaridou L. (2010). Size-adapted microcalcification segmentation in mammography utilizing scale-space signatures. Comput. Med. Imaging Graph..

[11] Ueda D., Kakinuma T., Fujita S., Kamagata K., Fushimi Y., Ito R., Matsui Y., Nozaki T., Nakaura T., Fujima N. (2024). Fairness of artificial intelligence in healthcare: Review and recommendations. Jpn. J. Radiol..

[12] Rana M.S., Shuford J. (2024). AI in Healthcare: Transforming Patient Care through Predictive Analytics and Decision Support Systems. J. Artif. Intell. Gen. Sci..

[13] Guerroudji M.A. (2017). Segmentation des Clichés Mammographiques en Vue de la Détection des Foyers de Micro-Calcifications Mammaires: Application à la Base de Données CHU de Tizi-Ouzou. Ph.D. Thesis.

[14] Jordan M.I., Mitchell T.M. (2015). Machine learning: Trends, perspectives, and prospects. Science.

[15] Rasool A., Bunterngchit C., Tiejian L., Islam M.R., Qu Q., Jiang Q. (2022). Improved machine learning-based predictive models for breast cancer diagnosis. Int. J. Environ. Res. Public Health.

[16] Desai M., Shah M. (2021). An anatomization on breast cancer detection and diagnosis employing multi-layer perceptron neural network (MLP) and Convolutional neural network (CNN). Clin. eHealth.

[17] Sidey-Gibbons J.A.M., Sidey-Gibbons C.J. (2019). Machine learning in medicine: A practical introduction. BMC Med. Res. Methodol..

[18] Obaid O.I., Mohammed M.A., Ghani M.K.A., Mostafa A., Taha F. (2018). Evaluating the performance of machine learning techniques in the classification of Wisconsin Breast Cancer. Int. J. Eng. Technol..

[19] Gilbert F.J., Pinker-Domenig K. (2019). Diagnosis and staging of breast cancer: When and how to use mammography, tomosynthesis, ultrasound, contrast-enhanced mammography, and magnetic resonance imaging. Diseases of the Chest, Breast, Heart and Vessels 2019–2022: Diagnostic and Interventional Imaging.

[20] Mandelbrot B.B., Mandelbrot B.B. (1982). The Fractal Geometry of Nature.

[21] Iranmakani S., Mortezazadeh T., Sajadian F., Ghaziani M.F., Ghafari A., Khezerloo D., Musa A.E. (2020). A review of various modalities in breast imaging: Technical aspects and clinical outcomes. Egypt. J. Radiol. Nucl. Med..

[22] Wolberg W.H., Mangasarian O.L. (1990). Multisurface method of pattern separation for medical diagnosis applied to breast cytology. Proc. Natl. Acad. Sci. USA.

[23] Cortes C., Vapnik V. (1995). Support-vector networks. Mach. Learn..

[24] Sivapriya J., Kumar A., Sai S.S., Sriram S. (2019). Breast cancer prediction using machine learning. Int. J. Recent Technol. Eng..

[25] Elsayad A.M. (2010). Predicting the severity of breast masses with ensemble of Bayesian classifiers. J. Comput. Sci..

[26] Ray S. Easy Steps to Learn Naive Bayes Algorithm with Codes in Python and R. *Anal. Vidhya 6*. https://www.datasciencecentral.com/6-easy-steps-to-learn-naive-bayes-algorithm-with-code-in-python/.

[27] Lowes S., Leaver A., Redman A. (2019). Diagnostic and interventional imaging techniques in breast cancer. Surgery.

[28] Chaaban R., Issa W., Bouakaz A., Zaylaa A.J. Hypertensive Disorders of Pregnancy: Kurtosis-Based Classification of Fetal Doppler Ultrasound Signals. Proceedings of the 2019 Fifth International Conference on Advances in Biomedical Engineering (ICABME).

[29] Küçük R. (2023). Forecasting foreign exchange rate with machine learning techniques. Master’s Thesis.

[30] McHugh M.L. (2012). Interrater reliability: The kappa statistic. Biochem. Medica.

[31] Renukadevi N., Thangaraj P. (2013). Performance evaluation of SVM–RBF kernel for medical image classification. Glob. J. Comput. Sci. Technol..

[32] Kharya S., Agrawal S., Soni S. (2014). Naive Bayes classifiers: A probabilistic detection model for breast cancer. Int. J. Comput. Appl..

[33] Street W.N., Wolberg W.H., Mangasarian O.L. Nuclear feature extraction for breast tumor diagnosis. Proceedings of the Biomedical Image Processing and Biomedical Visualization.

